# Complete resection of recurrent and initially unresectable dermatofibrosarcoma protuberans downsized by Imatinib

**DOI:** 10.1186/1477-7819-11-59

**Published:** 2013-03-08

**Authors:** Dennis A Wicherts, Frits van Coevorden, Houke M Klomp, Martine A van Huizum, J Martijn Kerst, Rick LM Haas, Hester H van Boven, JA van der Hage

**Affiliations:** 1 Department of Surgical Oncology, Netherlands Cancer Institute - Antoni van Leeuwenhoek Hospital, Plesmanlaan 121, Amsterdam, 1066CX, the Netherlands; 2 Department of Plastic and Reconstructive Surgery, Netherlands Cancer Institute - Antoni van Leeuwenhoek Hospital, Plesmanlaan 121, Amsterdam, 1066CX, the Netherlands; 3 Department of Medical Oncology, Netherlands Cancer Institute - Antoni van Leeuwenhoek Hospital, Plesmanlaan 121, Amsterdam, 1066CX, the Netherlands; 4 Department of Radiotherapy, Netherlands Cancer Institute - Antoni van Leeuwenhoek Hospital, Plesmanlaan 121, Amsterdam, 1066CX, the Netherlands; 5 Department of Pathology, Netherlands Cancer Institute - Antoni van Leeuwenhoek Hospital, Plesmanlaan 121, Amsterdam, 1066CX, the Netherlands

**Keywords:** Dermatofibrosarcoma protuberans, Imatinib, Surgery

## Abstract

Curative surgical treatment of recurrent, locally advanced dermatofibrosarcoma protuberans is often limited owing to a close relation of the tumor with important anatomical structures. Targeted therapy with imatinib, a tyrosine kinase inhibitor, may cause significant reduction of tumor volume, thereby enabling radical surgery. This treatment strategy, therefore, offers a chance of cure for selected patients with advanced dermatofibrosarcoma protuberans. In addition, preoperative treatment with imatinib may decrease possible disfigurement related to radical surgery for large tumors.

## Background

Dermatofibrosarcoma protuberans (DFSP), as a low- to intermediate-grade soft tissue sarcoma, is a locally aggressive tumor owing to its infiltrative growth pattern. Following initial resection, local recurrence is a common dilemma, while distant metastases usually are rare (5%) [1]. DFSP comprises around 2% to 6% of all soft tissue sarcomas and its incidence is almost double among black compared to white persons [2,3].

Diagnosis of DFSP is generally made by core needle or open biopsy. Immunohistochemical analysis (CD34 antigen expression) can be used in addition to histological tumor evaluation. In addition, magnetic resonance imaging is helpful in preoperative planning to evaluate the local extent of the tumor. Screening for distant metastases is performed by computed tomography imaging of the chest.

Radical surgical resection is the first choice of treatment of DFSP. Minimal tumor-free resection margins of 2 to 3 cm are recommended owing to the relatively high risk of local recurrence (up to 50%) [4-6]. However, such margins generally are difficult to obtain due to the anatomical location of the tumor. Postoperative radiotherapy is often administered to reduce the risk of local recurrence [7]. In cases of locally advanced tumor growth in close relation to vital structures, a multidisciplinary approach including reconstructive surgery is often needed.

For patients with unresectable disease, the application of targeted therapy with imatinib has recently been evaluated. In most DFSP patients (around 90%), a t(17;22) chromosomal translocation leads to upregulation of the platelet-derived growth factor B pathway, resulting in uncontrolled cell division [1]. Imatinib, originally developed to treat patients with chronic myelogenous leukemia, acts through inhibition of tyrosine kinase, thereby blocking the platelet-derived growth factor B (PDGFB) pathway and causing tumor cell apoptosis [1,8]. Results of imatinib treatment in patients with DFSP are encouraging but still limited. Some case series have recently been published, reporting favorable responses in patients with recurrent or metastatic disease [8,9]. Interestingly, the presence of a t(17;22) chromosomal translocation seems to be associated with the response to imatinib [10].

## Case presentation

A 47-year-old African man was referred to our clinic with a recurrent, locally advanced DFSP of the right periclavicular region. Primary resection of a DFSP in the same region was performed approximately six years earlier, followed by two additional resections for recurrent disease. Local radiotherapy (60 Gray) was administered after the last resection.

**Figure 1:**
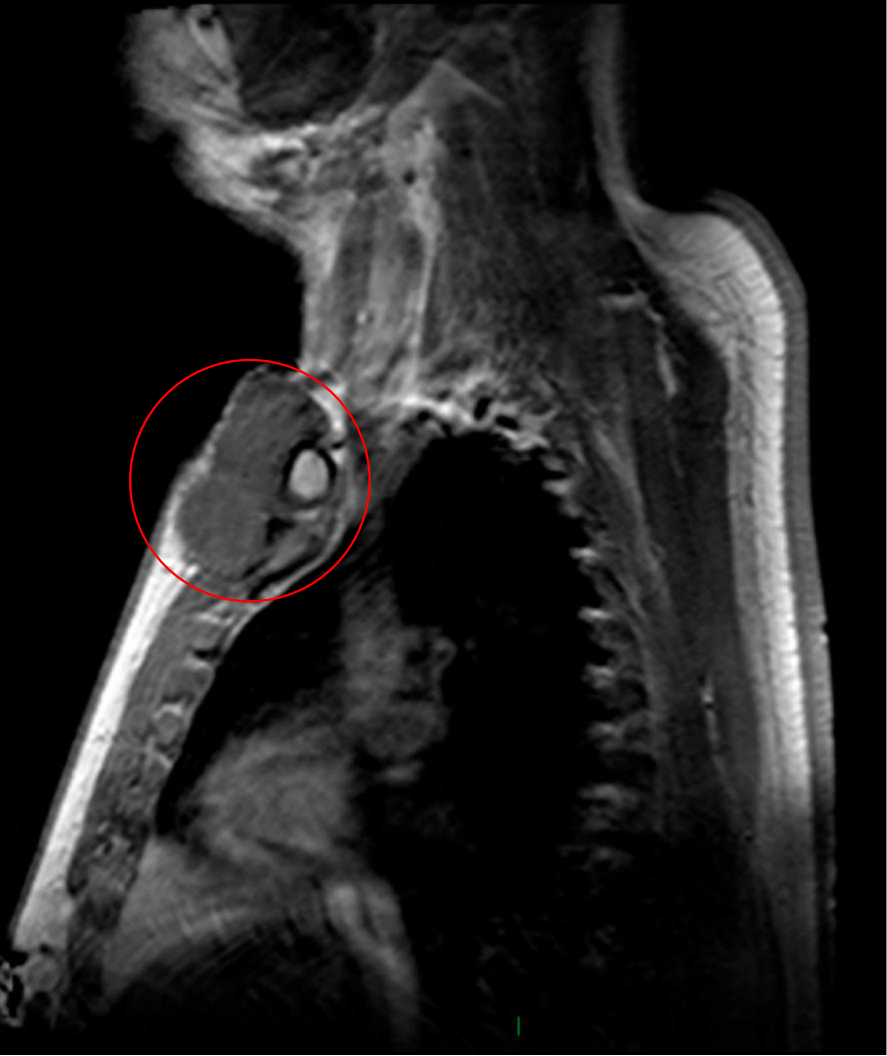
Magnetic resonance imaging showing the presence of locally advanced tumor growth with a close relation to the clavicula and internal jugular vein.

The current locally advanced tumor recurrence developed one year before the patient’s visit to our hospital. At physical examination, local signs of radiotherapy damage to the skin were noted in the right periclavicular region. Tumor was palpated underneath the clavicula as well as on the right side of the lower neck. Additional magnetic resonance imaging showed the presence of locally advanced tumor growth with a close relation to the clavicula and internal jugular vein, limiting the possibilities of radical surgery (Figure 1). No signs of distant metastases were demonstrated. Ultrasound core needle biopsy in the referring center suggested a high-grade soft tissue tumor, possibly fibrosarcoma, potentially due to malignant dedifferentiation of the original DFSP. Repeated biopsy in our clinic showed a low-grade mesenchymal tumor, positive for CD34, suggestive of DFSP. Fluorescence *in situ* hybridization (FISH) analysis revealed the presence of a t(17;22) chromosomal translocation, confirming the diagnosis of DFSP.

**Figure 2:**
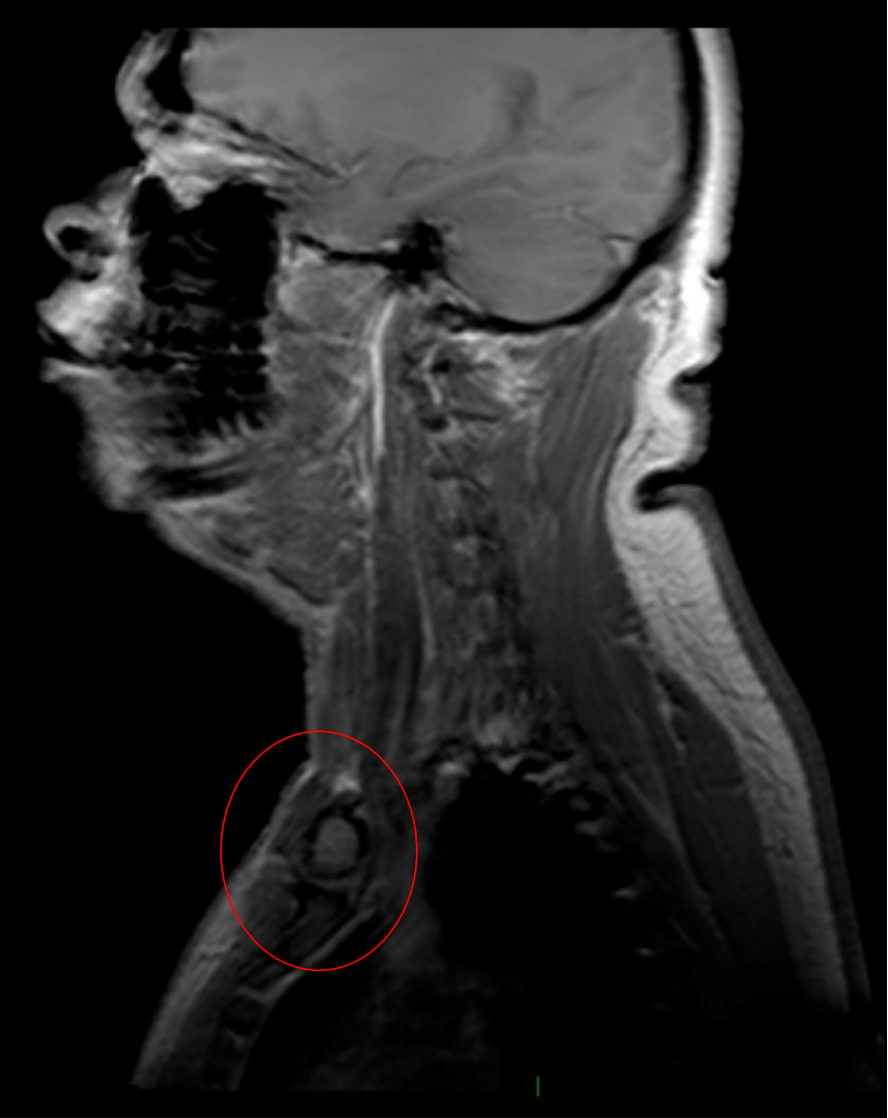
Magnetic resonance imaging showing partial tumor response after three months of imatinib treatment.

The patient was subsequently treated with imatinib 400 mg daily according to our institutional protocol for locally advanced gastrointestinal stromal tumors (GIST), resulting in a partial tumor response after three months of treatment (Figure 2). Assessment after six months showed stable disease and no signs of distant metastases. Due to the marked tumor response, radical surgery could now be performed without sacrificing vital structures. The tumor was resected en bloc with part of the underlying pectoralis major muscle, the periosteum of the clavicle and part of the sternocleidomastoid muscle. The internal jugular vein was free of tumor and, therefore, not sacrificed. Resection margins were macroscopically tumor-free. A plastic surgeon was consulted to close the defect by transposition of the pectoralis major muscle in combination with a split skin graft. The patient recovered without any complications.

The pathology report showed a radically resected low-grade mesenchymal tumor, with a near complete tumor response. Tumor-free margins were less than 1 mm. Immunohistochemical and RT-PCR analysis confirmed the diagnosis of DFSP. The lesion was positive for vimentin, aSMA and CD34, and negative for caldesmon, myoD1, CD117, desmin, NF, Cam5.2, pankeratin, Myf4 and S100 markers. RT-PCR sequencing was performed for C-KIT and PDGFR-alpha mutations to differentiate between GIST and DFSP. No mutations in exons 9, 11, 13 and 17 of the C-KIT gene were found nor were any mutations found in exons 12, 14 and 18 of the PDGFR-alpha gene.

The patient is currently followed at three month intervals by magnetic resonance imaging of the operated area. Computed tomography scanning of the chest will be performed annually. To reduce the risk of local recurrence, imatinib treatment will be continued for two years postoperatively.

## Conclusions

Although DFSP is commonly a low-grade tumor, approximately 15% of cases contain a high-grade sarcoma component. Also, high-grade dedifferentiation may occur in the course of the disease [11]. The patient described in our case presented with initially unresectable recurrent DFSP of the periclavicular region, with signs of high-grade dedifferentiation at core biopsy. Subsequently, his clinical response to imatinib was significant, resulting in the possibility of radical surgery. In addition, histological assessment of the resected tumor revealed a near complete tumor response. A positive relation between high-grade subtypes of DFSP and response to imatinib may, therefore, be suggested, emphasizing the efficiency of this targeted therapy in selected patients. A possible explanation of this relationship may be a higher incidence of the t(17;22) chromosomal translocation in patients with high-grade DFSP [11,12].

Taken together, our results strengthen the use of imatinib in downsizing initially unresectable DFSP, offering a chance of cure for selected patients following secondary surgery. In addition, for upfront resectable patients, preoperative treatment with imatinib may decrease possible disfigurement related to radical surgery for large tumors.

## Consent

No informed consent from the patient was considered necessary for publication of this case report since no details are presented that could lead to the identification of the patient.

## Abbreviations

DFSP: Dermatofibrosarcoma protuberans; GIST: Gastrointestinal stromal tumor; PDGFB: Platelet-derived growth factor B; RT-PCR: Reverse transcriptase polymerase chain reaction.

## Competing interests

The authors declare that they have no competing interests.

## Authors’ contributions

Conception and study design/data analysis: DW, FC, HK, MH, JK, RH, HB, JH. Manuscript drafting: DW, FC, HK, MH, JK, RH, HB, JH. Final approval of the manuscript: DW, FC, HK, MH, JK, RH, HB, JH. All authors read and approved the final manuscript.

## References

[B1] Rutkowski P Wozniak A Switaj T Advances in molecular characterization and targeted therapy in dermatofibrosarcoma protuberans Sarcoma 2011 2011 959132 21559214 10.1155/2011/959132 PMC3087969

[B2] Kransdorf MJ Malignant soft-tissue tumors in a large referral population: distribution of diagnoses by age, sex, and location AJR Am J Roentgenol 1995 164 129 134 10.2214/ajr.164.1.7998525 7998525

[B3] Criscione VD Weinstock MA Descriptive epidemiology of dermatofibrosarcoma protuberans in the United States, 1973 to 2002 J Am Acad Dermatol 2007 56 968 973 10.1016/j.jaad.2006.09.006 17141362

[B4] Chang CK Jacobs IA Salti GI Outcomes of surgery for dermatofibrosarcoma protuberans Eur J Surg Oncol 2004 30 341 345 10.1016/j.ejso.2003.12.005 15028319

[B5] Kimmel Z Ratner D Kim JYS Wayne JD Rademaker AW Alam M Peripheral excision margins for dermatofibrosarcoma protuberans: a meta-analysis of spatial data Ann Surg Oncol 2007 14 2113 2120 10.1245/s10434-006-9233-3 17468914

[B6] Rutgers EJ Kroon BB Albus-Lutter CE Gortzak E Dermatofibrosarcoma protuberans: treatment and prognosis Eur J Surg Oncol 1992 18 241 248 1607035

[B7] Haas RL Keus RB Loftus BM Rutgers EJ van Coevorden F Bartelink H The role of radiotherapy in the local management of dermatofibrosarcoma protuberans. Soft Tissue Tumours Working Group Eur J Cancer 1997 33 1055 1060 10.1016/S0959-8049(97)00026-9 9376187

[B8] Rubin BP Schuetze SM Eary JF Norwood TH Mirza S Conrad EU Bruckner JD Molecular targeting of platelet-derived growth factor B by imatinib mesylate in a patient with metastatic dermatofibrosarcoma protuberans J Clin Oncol 2002 20 3586 3591 10.1200/JCO.2002.01.027 12202658

[B9] Labropoulos SV Fletcher JA Oliveira AM Papadopoulos S Razis ED Sustained complete remission of metastatic dermatofibrosarcoma protuberans with imatinib mesylate Anticancer Drugs 2005 16 461 466 10.1097/00001813-200504000-00014 15746584

[B10] McArthur GA Demetri GD van Oosterom A Heinrich MC Debiec-Rychter M Corless CL Nikolova Z Dimitrijevic S Fletcher JA Molecular and clinical analysis of locally advanced dermatofibrosarcoma protuberans treated with imatinib: imatinib target exploration consortium study B2225 J Clin Oncol 2005 23 866 873 10.1200/JCO.2005.07.088 15681532

[B11] Abbott JJ Erickson-Johnson M Wang X Nascimento AG Oliveira AM Gains of COL1A1- PDGFB genomic copies occur in fibrosarcomatous transformation of dermatofibrosarcoma protuberans Mod Pathol 2006 19 1512 1518 16980946 10.1038/modpathol.3800695

[B12] Llombart B Monteagudo C Sanmartín O López-Guerrero JA Serra-Guillén C Poveda A Jorda E Fernandez-Serra A Pellín A Guillén C Llombart-Bosch A Dermatofibrosarcoma protuberans: a clinicopathological, immunohistochemical, genetic (COL1A1-PDGFB), and therapeutic study of low-grade versus high-grade (fibrosarcomatous) tumors J Am Acad Dermatol 2011 65 564 575 10.1016/j.jaad.2010.06.020 21570152

